# Association between social participation patterns and social adaptation among retired Tibetan immigrants: the mediating effect of institutional capital

**DOI:** 10.3389/fpubh.2024.1488356

**Published:** 2024-11-21

**Authors:** Yue Zhou, Menghe Ma, Sheng Sun

**Affiliations:** Department of Sociology, School of Law, Jiangnan University, Wuxi, China

**Keywords:** social participation, social adaptation, institutional capital, Tibetan immigrants, mediating effect

## Abstract

**Introduction:**

This study aims to examine the impact of various patterns of social participation on social adaptation among retired Tibetan immigrant older adults, as well as the mediating role of institutional capital in this relationship.

**Methods:**

A cross-sectional survey was conducted in Sichuan Province, China, involving 700 older adults who immigrated to Sichuan after retiring from Tibet. After excluding invalid samples, 501 were retained for the final analysis. Distinct patterns of social participation were identified using latent class analysis (LCA), and multiple regression models were employed to analyze the mediating role of institutional capital in the relationship between social participation patterns and social adaptation.

**Results:**

The study revealed four distinct patterns of social participation among the participants: full low-level participation, personal relationship-centric participation, social relevance-oriented participation, and balanced active participation. Institutional capital was found to play both a partial and a full mediating role in the relationship between social participation patterns and social adaptation.

**Discussion:**

These findings suggest that the social participation of retired immigrant seniors is influenced by the combined effects of role change, spatial transition, and aging, highlighting the urgent need to identify alternatives prior to integration into the local community to facilitate a smoother adaptation to life in their new environment.

## Introduction

Retirement immigrants from Tibet are autonomous migrants supported by national policies that consider the physical health challenges faced by the older adults in highland climates. This has led to the formation of a retirement migration group primarily centered in Sichuan and Chongqing. The social participation of retired immigrants is viewed as an indicator of their adaptation to life in their new locations. A study on the social participation patterns of older adults in China reveals that 57.2% of older adults exhibit low levels of participation, which correlates with poor social adaptation (1). It is commonly believed that retired immigrants encounter greater challenges in social participation and experience more significant adaptation issues due to the environmental changes and role dislocations they face. In our investigation of Tibetan retired immigrants, we discovered that the support provided by the ‘Tibetan Retirement Relocation Policy’ creates favorable conditions for social adaptation (2). Additionally, the ‘two-track pension system’ has resulted in varying degrees of social adaptation among different categories of retired immigrants. This raises important questions: What social participation patterns are established by Tibetan retired immigrants? What is the correlation between the institutional capital represented by the type of working unit and the trajectory of Tibetan retired migrants from social participation to social adaptation? To address these questions and enhance the theoretical understanding of healthy aging, this study focuses on the Tibetan retired immigrant group, which is predominantly located in specific settlement areas, and investigates the relationship between their social participation patterns and social adaptation, with institutional capital serving as a mediating factor.

Social participation and social adaptation among immigrant older adults are often perceived as mutually reinforcing relationships, both behaviorally and psychologically. Social participation refers to an individual’s engagement in activities that involve interaction with others within a society or community (3). In contrast, social adaptation pertains to the reciprocal harmonization between the individual and their environment, emphasizing the relationship between the individual and society. This concept is often described as a “socialization process in spatial transition” and has been utilized in immigrant studies as an indicator of evaluating for the ability to establish a new life in the place of relocation and the state of societal well-being (2, 4, 5). Generally, it is acknowledged that the degree of social participation among older adults correlates with the extent of social adaptation; specifically, a higher level of social participation is associated with improved social adaptation status (3). A study examining migrating older adults in Korea revealed that mobility itself is a manifestation of active living, with migrating older adults exhibiting higher levels of social participation and life satisfaction (6). Furthermore, additional research has indicated that the characteristics of migrating older adults (such as health status and income) and the external social environment (including cultural differences and social acceptance) can constrain their patterns of social participation (7–9).

Unfortunately, existing studies often focus on examining the relationship between specific types of social participation activities or the overall level of social participation and social adaptation, neglecting the diverse and simultaneous dimensions of older adults’ social engagement. For instance, older adults may concurrently participate in various social activities, including community organizing, volunteering, and caring for grandchildren. However, different interrelationships between these patterns of social participation exist, and these relationships can significantly impact the lives and well-being of older individuals, particularly regarding social adaptation. Evidence suggests that social participation that emphasizes personal life or achieves a balance between personal and family life is more conducive to social adaptation than family-centered social participation (1). Nonetheless, the relationship between social participation patterns and social adaptation may be more complex in certain age groups, such as the Tibetan immigrant older adults, due to differences in social participation behaviors, psychological adaptation, and cultural identity (10). Therefore, it is essential to explore the patterns and characteristics of social participation to gain insights into the relationship between social participation and social adaptation among older migrant groups.

Social capital influences migrants’ choices, shapes their lives in the places of immigration, and a lack of social capital increases the risk of social adaptation challenges (11–13). Social capital, which can provide value and benefits, is categorized into relational and institutional capital (14). Social stratification leads to disparities in resources that affect migration decisions and social adaptation outcomes (15, 16). Currently, the impact of relational capital on social adaptation is predominantly studied in the context of older migration, with research variables including social networks, social participation, social trust, social support, and community environment (17–20). Institutional capital is primarily examined in relation to quality of life and social adaptation following labor migration. For instance, research has explored the effects of Italian migrant subsidies on the income and migration decisions of individuals with varying education levels (16). Additionally, a study of Chinese migrant workers demonstrated that the accumulation of social capital enhances their social adaptation capabilities (21). Researchers have increasingly acknowledged that the effects of social capital are long-term; a study on rural–urban migrants in China revealed that the mental health of the migrating population was bolstered by early (7–15 years prior) social capital (22).

When examining the relationship between social participation and social adaptation of migrating older adults in China, it is essential to consider the variations in institutional capital resulting from retirement units. Currently, the primary source of income for most Chinese retirees remains their pension. Although the influence of the unit system on the social lives of active workers has gradually diminished, the traditional “working unit system” has progressively transitioned to a market economic system (23). Nevertheless, the type of unit continues to exert a long-term impact on retired workers in China. Although retired migrants may experience a loss of social status and relational capital associated with their pre-retirement unit membership, they remain subject to the “double-track pension system” (24). This system categorizes employee pensions into two main types: institutional and enterprise. The disparities in contribution bases, funding sources, and responsible parties for payments result in social stratification based on the type of retirement unit. In the Tibet Autonomous Region (TAR), in addition to the “two-track pension system,” there exists an additional disparity concerning the resettlement benefits for retired workers who have been relocated (25).

As mentioned above, existing research has thoroughly demonstrated the positive impact of social participation on social adaptation, as well as the role of social capital in facilitating this adaptation among immigrant populations and older immigrants. While studies have begun to delineate social participation patterns among seniors, the specific patterns of social participation among retired immigrant older adults remain under-explored. Given the significant correlations and predictive effects among these variables, there is a pressing need to investigate the unique social integration practices and institutional identities of retired older Tibetans. This necessitates an in-depth exploration of how the social participation patterns of Tibetan retired immigrant older adults influence their social adaptation, along with the construction of a theoretical model that clarifies the relationship between these factors. Consequently, this study aims to examine the potential mediating role of institutional capital, influenced by the type of working unit, in the relationship between social participation patterns and social adaptation. This endeavor seeks to uncover the complex pathways linking social participation profiles to the adaptation experiences of Tibetan retired older adults, as well as to identify the potential risks and benefits associated with institutional capital. Based on the existing literature, this study proposes the following three research hypotheses.

*H1*: There are several different social participation patterns among Tibetan retired immigrant older adults.

*H2*: Distinct social participation patterns have difference correlations with social adaptation. Social Relationship-orientated and high-level participatory pattern is beneficial for social adaptation. Conversely, individual or family-centered and low-participation patterns may be negatively associated with social adaptation.

*H3*: The institutional capital plays a mediating role in the relationship between distinct social participation patterns and social adaptation.

## Materials and methods

### Sample and data collection

This study conducted a cross-sectional survey of retired Tibetan older individuals who had relocated from Tibet to various provinces. A purposive sampling method was employed, targeting older adults aged 60 years and above who could complete the questionnaire independently. Individuals who lacked knowledge or faced limitations in daily activities or cognitive abilities were excluded from the study. The questionnaire survey was administered by trained enumerators via telephone interviews. Respondents’ contact details were provided by the authorities responsible for the resettlement of Tibetan migrant populations. An invitation text message explaining the research topic, objectives, and methodology was sent to all retired relocated individuals through these authorities, inviting them to participate in the survey while ensuring the anonymity and confidentiality of the process. The survey was conducted over a two-month period, from January 25 to March 28, 2024. A total of 700 questionnaires were distributed, and 568 were returned, resulting in a recovery rate of 81.1%. After excluding 21 invalid questionnaires—where more than half of the questions were unanswered or the responses were highly repetitive—and 46 samples with missing information on social participation, a total of 501 questionnaires were deemed suitable for statistical analysis. The study received approval from the ethics committee, participants provided informed consent, and all data were anonymized. Participant characteristics are detailed in Appendix Table 1.

### Measurement

#### Social participation pattern

This study assessed the social participation of retired Tibetan immigrant older adults, encompassing activities such as visiting family and friends, playing games like mahjong, chess, and cards, and utilizing community spaces. It also included aiding with relatives, friends, or neighbors with whom they do not reside (e.g., caring for grandchildren, performing household chores), engaging in personal outdoor activities (such as square dancing, exercising, and practicing qigong), and participating in social organizations (e.g., hobby clubs, political parties, trade unions). Additionally, the study considered volunteering or charitable activities, caring for sick or disabled individuals not residing with them, and economic activities like stock speculation and odd jobs. Participants were also involved in attending hobby classes, training courses, or senior universities, as well as using the Internet and social software (including interest clubs, party activities, and labor union activities). Participants were asked to indicate whether they had engaged in these social activities over the past three months, with a score of 1 assigned for participation and 0 for non-participation.

#### Social adaptation

To measure social adaptation, this study employed the brief version of the Social Adaptation Scale developed by Yang (10). The scale is divided into three dimensions (KMO = 0.812, *p* < 0.000): psychological adaptation, behavioral adaptation, and cultural adaptation. Respondents rated the content of each social capital statement (e.g., ‘I like the city/place where I live now,’ ‘In my daily life, I follow local customs,’ ‘I live locally, but I always feel that I am an outsider,’ etc.) on a 6-point rating scale, with 1 indicating strong disagreement and 6 indicating strong agreement. The analyses utilized the mean score to assess the degree of social adjustment (out of 6), with higher scores reflecting better social adjustment. The overall reliability of Cronbach’s *α* for social adjustment was 0.77.

#### Institutional capital

Institutional capital was assessed using a single item question: ‘What is the nature of your retirement unit?’ Currently, there is no universally accepted instrument for measuring institutional capital in China. Given the dual-track pension system, which is a significant factor contributing to social stratification among retired individuals in China, the system operates by differentiating contributions and payments based on the type of work unit. Furthermore, the Tibet Autonomous Region has introduced variations in resettlement benefits for retired individuals who have been relocated, adding another layer to the ‘two-track pension system.’ Consequently, the type of retirement unit establishes a hierarchy of institutional capital that effectively reflects an individual’s institutional capital status. Respondents are asked to select the option that corresponds to their unit situation: enterprises (assigned a value of 1), institutions (assigned a value of 2), or government agencies (assigned a value of 3). The scoring system ranges from 1 to 3, with higher scores indicating greater institutional capital.

#### Control variables

The control variables in this study included participants’ age, gender, level of education (measured in years), living arrangements, region of residence, and self-rated health. Gender was treated as a binary variable, with male coded as 0 and female as 1. Age and level of education were continuous variables. Living arrangements were categorized as either living alone or not, with living alone assigned a score of 1 and not living alone a score of 0. The region of residence consisted of categorical variables classified into three categories: rural (coded as 1), urban (coded as 2), and metropolitan (coded as 3). Self-rated health status was assessed using a 5-point Likert scale, with 1 indicating very poor health and 5 indicating very good health.

### Analytic strategy

The data analysis comprised two stages. In the first stage, a Latent Class Analysis (LCA) was employed to identify patterns of social participation among older Tibetan immigrants. A series of latent class models were constructed using an exploratory stepwise additive approach based on ten types of social participation activities. The optimal number of latent classes, representing distinct patterns of social support, was determined by comprehensively comparing the fit indices of each model, including AIC, BIC, adBIC, entropy, and VLRT. Smaller values of AIC, BIC, and adBIC indicate a better model fit, while statistically significant values of VLRT suggest that the K-model provides a superior fit compared to the K-1 model. An entropy value close to 1 signifies higher classification accuracy (26). Following the classification of social participation among retired Tibetan immigrant older adults, the second stage involved employing a regression model to analyze the impact of institutional capital on the social adaptation of these individuals, as well as the mediating role of the type of social participation. The significance of the mediating effect was subsequently assessed using the Sobel test. Statistical analyses were performed using STATA 14 and Mplus 8.0 software.

## Results

### Latent class analysis for social participation pattern

**Figure 1:**
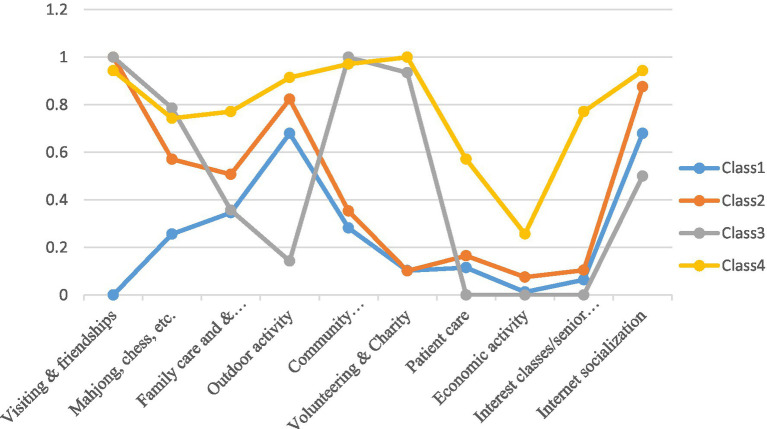
Form of three latent classes by social participance item.

After a comprehensive evaluation of the model fit indices, the optimal number of subgroups for retired Tibetan immigrant older individuals was determined to be four (see Table 1). This conclusion was based on the relatively low values of the Akaike Information Criterion (AIC) and Bayesian Information Criterion (BIC), entropy values approaching 1, and the significance of the Vuong-Lo–Mendell–Rubin likelihood ratio test (VLBT) for the four-class model. As illustrated in Figure 1, four distinct models of social participation were identified based on the participants’ profiles. Class 1, which we termed ‘full low-level participation,’ comprises 16.5% (*N* = 78) of the sample. This group exhibited a low overall level of social participation, primarily engaging in outdoor physical exercise and Internet surfing, and is characterized as a high-risk group for social alienation. The second class, ‘personal relationship-centric participation’ (Class 2), includes 73.1% (*N* = 345) of the retired Tibetan immigrants, representing most of the sample. In contrast to Class 1, this group prioritized personal relationships, such as socializing with friends and visiting family, and demonstrated greater activity in various other pursuits, including increased engagement in online social interactions. Class 3, designated as ‘social relevance-oriented participation,’ consists of 14 individuals (3%) who exhibit a strong sense of social responsibility. This group maintains their personal relationships and is actively involved in community activities, volunteer work, and online socialization. Class 4 represented 7% (*N* = 35) of the total sample and was designated as balanced active participation. This class exhibits a high level of engagement in various social activities, with the exception of relatively lower involvement in stock speculation and odd jobs. It falls within the active group of retired migrants. This finding suggests that there are diverse patterns of social participation among Tibetan retired immigrant older individuals in China.

**Table 1:** Latent class model fit.

		Latent classes				
		2	3	4	5	6
Model fit index	AIC	5038.202	4996.833	4989.963	4987.537	4994.636
	BIC	5126.793	5131.828	5171.363	5215.341	5268.845
	adBIC	5060.137	5030.257	5034.878	5043.941	5062.530
	VLRT^+^	−2555.67^***^	−2498.10^***^	−2466.42^***^	−2451.98	−639.046^***^
	Entropy	0.610	0.872	0.889	0.911	0.784
Sub-group, *N* (%)	1	47 (9.9)	360 (76.3)	78 (16.5)	40 (8.5)	26 (6.3)
	2	425 (90.1)	34 (7.2)	345 (73.1)	36 (7.6)	14 (3.8)
	3		78 (16.5)	14 (3.0)	310 (65.7)	8 (1.6)
	4			35 (7.4)	70 (14.8)	80 (17.3)
	5				16 (3.4)	319 (65.5)
	6					25 (6.0)

*p < 0.05, **p < 0.01, ***p < 0.001. +: Ho = k-1 class; Loglikelihood Difference (P).

### Regression analysis and moderation effects

A multiple regression analysis model was employed to investigate the mediating effect of institutional capital on the support patterns influencing the social adaptation of retired Tibetan migrant older adults. In this mediation effect analysis model, the independent variable (social participation pattern) was coded as a dummy variable, while both the mediating variable (institutional capital) and the dependent variable (social adaptation) were treated as continuous variables. The tolerance levels for all variables exceeded 0.1 (ranging from 0.609 to 0.961), and the Variance Inflation Factor (VIF) values were below 10 (ranging from 1.046 to 1.643), indicating that the regression model was free from multicollinearity.

The results of the regression analysis are presented in Table 2. First, the analyses indicate that patterns of partial social participation significantly affect institutional capital, even after controlling for other potential influences (Path a). Compared to the full low-level participation (class 1), social relevance-oriented participation (class 3, *B* = 0.422, *p* = 0.015) demonstrates a higher level of institutional capital, while the balanced active type (class 4, *B* = −0.347, *p* = 0.022) shows a lower level of institutional capital. In contrast, the effect of personal relationship-centric participation (class 2, *B* = 0.030, *p* = 0.192) is not statistically significant. Second, in Path b, institutional capital has a significant positive effect on social adjustment (*B* = 0.320, *p* = 0.000), suggesting that higher institutional capital is associated with better social adjustment among immigrant older individuals. Finally, in Path c’, both class 2 (*B* = 0.394, *p* = 0.001) and class 3 (*B* = 0.437, *p* = 0.048) positively influence the social adjustment of retired Tibetan immigrant older adults. However, balanced active participation (class 4, *B* = 0.361, *p* = 0.092) does not have a statistically significant effect on the social adjustment of immigrant older adults (see step 1). When considering both social participation patterns and institutional capital (see step 2), the influence of the Personal Interaction type on the social adjustment among immigrant seniors is slightly reduced (roughly 5%). The influence of class 3 decreases by approximately 7% and becomes non-significant. Additionally, the influence of class 4 increases by about 8% and is significant.

**Table 2:** Multiple regression analysis among variables influencing social adaptation and mediating effect.

Variables	Institutional capital (Path a)			Social adaptation (Path b)			Social adaptation (Path c)					
							Step 1			Step 2		
	*B*	t	S. E.	*B*	t	S. E.	*B*	t	S. E.	*B*	t	S. E.
Constant	3.731	5.653^***^	0.660	3.449	5.594^***^	0.616	2.521	3.782^***^	0.667	3.184	4.301^***^	0.740
Control variables^a^	-			-			-			-		
SPP (Ref. C1)												
C2	−0.030	−0.347	0.086				0.394	3.131^**^	0.126	0.375	2.886^**^	0.130
C3	0.422	2.331^*^	0.181				0.437	1.978^*^	0.221	0.407	1.346	0.303
C4	−0.347	−2.115^*^	0.164				0.361	1.742	0.207	0.388	2.036^*^	0.191
IC				0.320	3.919^***^	0.064				0.428	4.413^***^	0.097
F	45.127^***^			3.254^***^			3.519^***^			3.705^***^		
R^2^	0.146			0.176			0.196			0.244		

*p < 0.05, **p < 0.01, ***p < 0.001. a: Control variables have been put into the analytical model, including age, sex, education level, monthly income, self-rated health stats, living arrangement, marital status, residence region. SPP, social participation patterns; IC, institutional capital; SA, social adaptation; CI, full low-level participation; C2, personal relationship-centric participation; C3, social relevance-oriented participation; C4, balanced active participation.

**Figure 2:**
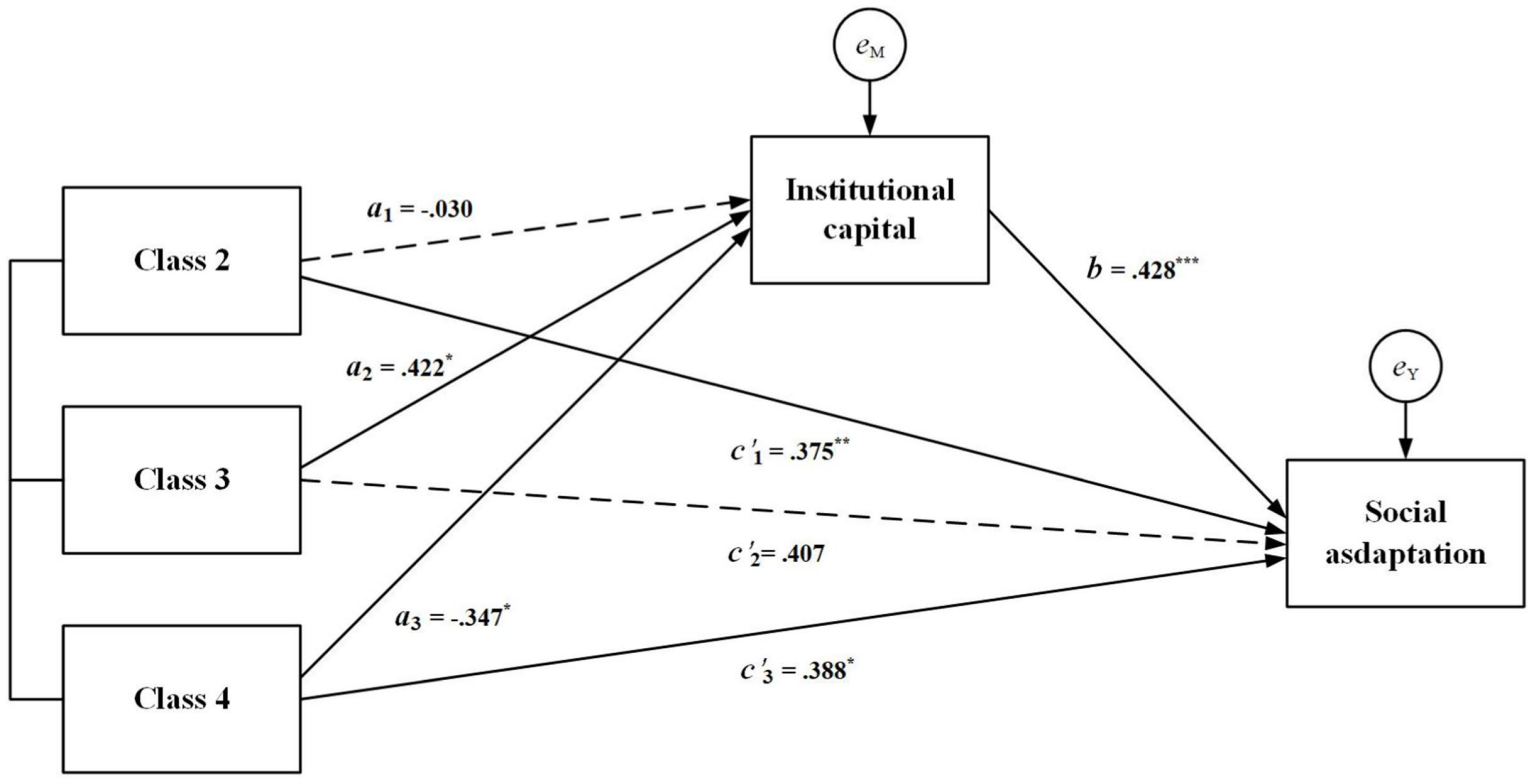
Mediating effect model.

To further investigate the mediating effect of institutional capital on the relationship between social support patterns and social adaptation, the Sobel test method was employed Lacobucci et al. (27). The results of the Sobel test are presented in Table 3. When considering the full low-level participation, the mediating effect of the pathway from personal relationship-centric participation (class 2) to institutional capital and subsequently to social adaptation was not statistically significant (*z* = −0.366, *p* = 0.712). However, institutional capital demonstrated a significant mediating effect (*z* = 1.967, *p* = 0.046) between social relevance-oriented participation (class 3) and the social adaptation of immigrant older adults. With the inclusion of the mediating variable, the direct effect of class 3 on the social adaptation of older immigrants was not significant (*B* = 0.371, *p* = 0.113), indicating that institutional capital fully mediated this relationship. A statistically significant mediating effect of institutional capital was also observed in the pathway from balanced active participation (class 4) to social adaptation (*z* = −2.254, *p* = 0.019). Furthermore, the direct effect of class 4 on the social adaptation of older immigrants was significant (*B* = 0.356, *p* < 0.05), suggesting that institutional capital partially mediates the relationship between class 4 and social adaptation. The overall profile of the mediating effects is illustrated in Figure 2.

**Table 3:** Moderating effect by Sobel test.

Paths (reference class 1)	Test statistic	S.E.
Class 2 → Institutional capital → Social Adaptation	−0.366	0.033
Class 3 → Institutional capital → Social Adaptation	1.967^**^	0.064
Class 4 → Institutional capital → Social Adaptation	−2.254^**^	0.058

^**^p < 0.05. Class 1 = Full low-level participation; Class 2 = Personal relationship-centric participation; Class 3 = Social relevance-oriented participation; Class 4 = Balanced active participation.

## Discussion

This study aims to examine the social participation patterns among retired Tibetan immigrants and their impact on social adaptation, as well as the mediating role of institutional capital, within the context of the interaction between individualization and aging trends. Based on the tendency of social participation, this research employs a latent class model to categorize the social participation patterns of retired Tibetan immigrants into four distinct types: full low-level participation, personal relationship-centric participation, social relevance-oriented participation, and balanced active participation. The proportions of each participation pattern are 16.5, 73.1, 3, and 7.4%, respectively, with all patterns exhibiting a high level of online social participation. Notably, personal relationship-centric participation dominates, contrasting sharply with the overall low social participation observed among the older adults in China. From the perspective of spatial shifts in the lives of migrating populations, retired Tibetan immigrants demonstrate spatial aggregation, significantly influenced by their pre-retirement social networks. Previous studies indicate that their primary contacts post-migration remain ‘colleagues with work experience in Tibet’ (25, 28, 29). However, the connection between retired immigrants and the host society proves to be challenging. The Sichuan-Chongqing region, the primary destination for Tibetan retirees, has historically served as a transition zone between Tibet and the broader world, exhibiting a high degree of territorial adaptation. Qualitative interviews further reveal that deeper engagement in the social life of their new location remains a significant challenge for Tibetan retirees (2).

Considering the institutional capital gradients caused by differences in the type of retirement unit, this was analyzed as a mediating variable. The findings reveal that institutional capital mediates the relationship between social participation patterns and the social adaptation of immigrant seniors. Firstly, it was observed that, compared to Full Low-level Participation, Personal relationship-centric participation (Class 2) and balanced active participation (Class 4) positively impact the social adaptation of retired Tibetan immigrant older adults, leading to enhanced social adjustment. Institutional capital amplifies the positive effects of Class 4 on social adjustment and partially mediates this relationship. Secondly, while social relevance-oriented participation does not exhibit a significant direct effect, it serves as a critical distal factor, with its impact fully mediated by institutional capital. This indicates that there are conditional differences in how social participation positively influences social adjustment. Variations in institutional capital result in differing levels of migration articulation among Tibetan retired immigrants, affecting aspects such as housing, income, services, and community engagement (25). The favorable institutional security provided by various organizations may inadvertently diminish their motivation for social participation, leading them to prioritize personal lives over social interactions. Conversely, in contexts where pensions are significantly lower than their pre-retirement incomes and where there is inadequate protection regarding housing and other security post-migration, these retired migrants tend to engage more actively in social activities within their new locales. They seek ‘localized’ solutions to the challenges of aging after migration, such as accessing information on public services and receiving social support from local residents and institutions (2).

This study has several limitations that should be addressed in future research. First, there are notable differences between Tibetan retirement immigrants and retirement immigrants in China more broadly. The Tibetan retired migrant community has emerged as a historical consequence of policies influenced by the restrictive household registration system and has expanded in both number and scope within a relatively concentrated community. In contrast, many retired migrants in China lack well-established communities, necessitating further verification of the applicability of this study’s findings. Second, this study focused on retired Tibetan migrants predominantly resettled through government relocation and employed purposive sampling to survey immigrant seniors in Sichuan Province, the primary site for government resettlement. Consequently, the findings may exhibit limited generalizability. Specifically, these results may not be applicable to all older migrant adults, necessitating caution in their interpretation and generalization. Given the diversity among older adults both in China and globally, further validation is essential before extending the findings to other groups, such as diaspora immigrants. Future research will seek to broaden the geographical scope of the survey and enhance the diversity of the sample to strengthen the validity of the generalized findings. Third, this study employed a regression model-based approach to analyze the mediating effects of multiple categories of independent variables. While this method enhances model stability and allows for the inclusion of multiple covariates simultaneously to mitigate the impact of confounding variables on the reliability of the results, it also has inherent limitations. For instance, all variables in the regression model are treated as significant, which may lead to an underestimation of the mediation effect due to potential measurement errors. Furthermore, the constraints of using regression models to assess mediation effects preclude the testing of equivalent models. Considering the numerous advantages of structural equation modeling in analyzing mediation models, future research should explore the use of structural equation modeling to investigate the mediation effects of multiple categories of independent variables in order to overcome these limitations. Fourth, while this study considered institutional capital as a mediating variable, there may be additional mediating or moderating factors to explore, such as external influences like social trust, digital life, community environment, and social support. However, due to the limitations of the survey data utilized in this study, it was not feasible to incorporate these variables into the analytical model for verification. Consequently, future research could broaden the investigation of factors influencing social adaptation among retired Tibetan immigrant older adults and refine the theoretical model, thereby providing evidence to enhance the well-being of seniors. Finally, this study utilized cross-sectional data, which limited the ability to ascertain causal relationships among the variables. Specifically, establishing the temporal order among the independent, mediating, and dependent variables presented challenges. To address this limitation and enhance the reliability of the findings, future studies could employ longitudinal data analysis and utilize randomized control groups to validate the causal connections among the variables. These approaches would facilitate a more nuanced analysis of how patterns of social participation evolve during retirement migration and their relationship with social adaptation.

## Conclusion

Social participation is recognized as a key indicator of how retired migrants adapt to life in a new environment. Retired Tibetan migrants face significant challenges in social participation and encounter more severe difficulties in social adaptation due to environmental changes, role mismatches, and cultural disparities. This study focuses on the retired Tibetan immigrant population to examine their social participation patterns and their association with social adaptation, as well as the mediating role of institutional capital in this relationship. The results indicate that the social participation patterns among retired Tibetan immigrants are diverse, revealing four distinct types of social participation. The ‘personal relationship-centric participation’ and ‘balanced active participation’ positively influence social adaptation. Furthermore, institutional capital serves as a mediating factor between social participation patterns and social adaptation. It can be concluded that social participation and adaptation have led to a distinct separation among contemporary retired immigrants. This phenomenon is fundamentally different from the gradual withdrawal from social engagement typically observed with aging; instead, it reflects the social isolation experienced by non-labor force migrants (30). The social participation of retired immigrants is influenced by the combined effects of role change, spatial transition, and aging (31). To adapt to life in their new location more effectively, there is an urgent need for these individuals to explore alternatives before fully integrating into the local community. Online socialization can somewhat mitigate the disruption of interpersonal relationships caused by spatial changes, as online platforms and communities offer valuable information that aids in adjusting to local life (32, 33). Furthermore, while the influence of China’s unit system has diminished, it continues to function as a form of institutional capital that significantly impacts the transition of retired individuals from social participation to social adaptation (34). Our findings indicate that Tibetan retired migrants from institutional backgrounds are more inclined to adopt a social participation approach centered on their personal lives, a tendency linked to the increased institutional capital support they receive post-retirement. This institutional capital enhances the migration adaptation capabilities of retired migrants, as it often correlates with greater access to disposable income, resources, information, and social support (20, 29). Such access facilitates a smoother adaptation process for this group of retired migrants in their post-migration lives.

## Data Availability

The data that support the findings of this study are available on request from the corresponding author. The data is not publicly available due to their containing information that could compromise the privacy of research participants.

## Appendix

**Table A1:** Participants’ socio-demographic profile.

Variables		*N* (%)/Mean (S.D.)
Age		62.79 (7.47)
Sex	Male	257 (51.3)
	Female	244 (48.7)
Educational level	Middle school and below	131 (26.2)
	High School	175 (35.0)
	University and above	194 (38.8)
Monthly income	Less than 5,000 CNY	101 (21.4)
	5,000–10,000 CNY	208 (44.0)
	10,001–15,000 CNY	127 (26.8)
	More than 15,000 CNY	37 (7.8)
Marital status	Divorced/widowed/unmarried	64 (12.8)
	Married	435 (87.2)
Live arrangement	Not living alone	415 (88.3)
	living alone	55 (11.7)
Residence region	Rural	20 (4.0)
	Urban	169 (24.0)
	Metropolitan	309 (62.0)
Self-rated health		3.17 (0.87)
Pre-retirement workplace	Enterprise	184 (38.3)
	Institutions/Government	296 (61.7)
social adaptation		4.85 (0.87)
Total		501 (100)

The results of the descriptive analyses for certain variables may be inconsistent with the total sample size due to the presence of missing values.
